# 3-(4-Bromo­phen­yl)-1-butyl-5-[1-(2-chloro-6-methyl­phen­yl)-1*H*-tetra­zol-5-yl]imidazolidine-2,4-dione

**DOI:** 10.1107/S1600536813016000

**Published:** 2013-06-15

**Authors:** Gabriel B. Hall, Federico Medda, Sue A. Roberts, Christopher Hulme

**Affiliations:** a Department of Chemistry and Biochemistry, University of Arizona, 1306 E University Blvd, Tucson, AZ 85721, USA; b BIO5 Oro Valley, College of Pharmacy, University of Arizona, 1580 E. Hanley Blvd, Oro Valley, AZ 85737, USA

## Abstract

In the title mol­ecule, C_21_H_20_BrClN_6_O_2_, the chloro-substituted benzene ring forms a dihedral angle of 77.84 (7)° with the tetra­zole ring and the bromo-substituted ring forms a dihedral angle of 43.95 (6)° with the imidazole ring. The dihedral angle between the tetra­zole and imidazole rings is 67.42 (8)°. The terminal methyl group of the butyl substituent is disordered over two sets of sites, with refined occupancies 0.67 (3) and 0.33 (3). In the crystal, there is a short Br⋯N contact of 3.183 (2) Å.

## Related literature

For the biological activity of imidazoline-2,4-diones, see: Thenmozhiyal *et al.* (2004 ▶); Brazil & Pedley (1998 ▶); Luer (1998 ▶); Matzukura *et al.* (1992 ▶); Knabe *et al.* (1997 ▶); Somsák *et al.* (2001 ▶); Moloney *et al.* (2001 ▶); Moloney *et al.* (1999 ▶); Sutherland & Hess (2000 ▶). For information on 1-5-disubstituted tetra­zoles. see: Al-Hourani *et al.* (2011 ▶); Brazil & Pedley (1998 ▶); Davulcu *et al.* (2009 ▶); Herr (2002 ▶); Quan *et al.* (2003 ▶); Van Poecke *et al.* (2011 ▶).
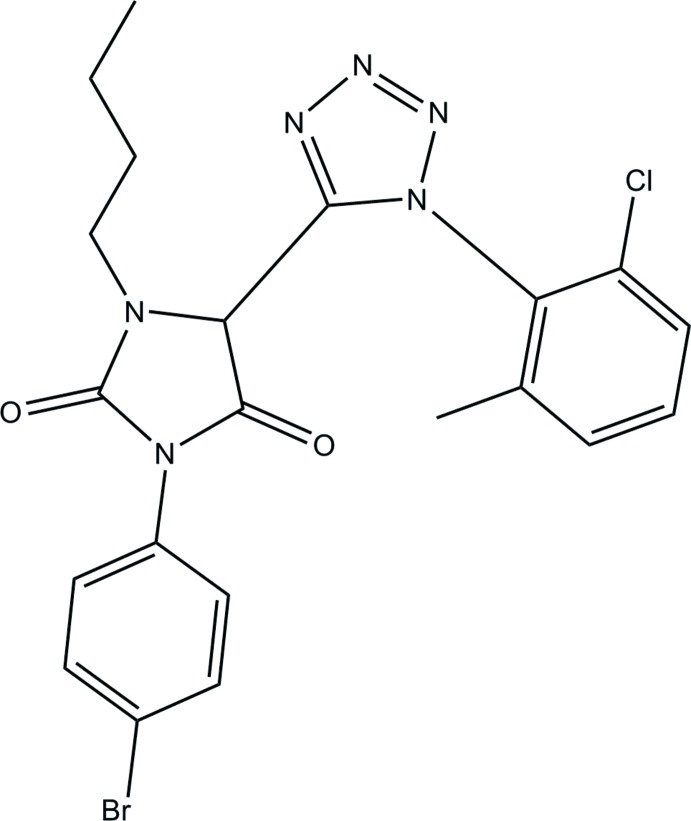


## Experimental

### 

#### Crystal data

C_21_H_20_BrClN_6_O_2_
*M* *_r_* = 503.79
Monoclinic, 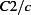

*a* = 27.9412 (9) Å
*b* = 8.8675 (3) Å
*c* = 19.6581 (6) Å
β = 112.500 (1)°
*V* = 4499.9 (3) Å^3^
*Z* = 8
Mo *K*α radiation
μ = 1.98 mm^−1^
*T* = 100 K
0.36 × 0.2 × 0.03 mm

#### Data collection

Bruker APEXII DUO CCD diffractometer
Absorption correction: multi-scan (*SADABS*; Bruker, 2009 ▶) *T*_min_ = 0.613, *T*_max_ = 0.746
79112 measured reflections
5647 independent reflections
4475 reflections with *I* > 2σ(*I*)
*R*_int_ = 0.043

#### Refinement

*R*[*F* ^2^ > 2σ(*F* ^2^)] = 0.037
*wR*(*F* ^2^) = 0.100
*S* = 1.03
5647 reflections
281 parameters
12 restraints
H-atom parameters not refined
Δρ_max_ = 1.34 e Å^−3^
Δρ_min_ = −1.07 e Å^−3^

### 

Data collection: *APEX2* (Bruker, 2009 ▶); cell refinement: *SAINT* (Bruker, 2009 ▶); data reduction: *SAINT*; program(s) used to solve structure: *SHELXS97* (Sheldrick, 2008 ▶); program(s) used to refine structure: *SHELXL97* (Sheldrick, 2008 ▶); molecular graphics: *OLEX2* (Dolomanov *et al.*, 2009 ▶); software used to prepare material for publication: *publCIF* (Westrip, 2010 ▶).

## Supplementary Material

Crystal structure: contains datablock(s) global, I. DOI: 10.1107/S1600536813016000/lh5623sup1.cif

Structure factors: contains datablock(s) I. DOI: 10.1107/S1600536813016000/lh5623Isup2.hkl

Supplementary material file. DOI: 10.1107/S1600536813016000/lh5623Isup3.cml

Additional supplementary materials:  crystallographic information; 3D view; checkCIF report

## Figures and Tables

**Fig. 1.**
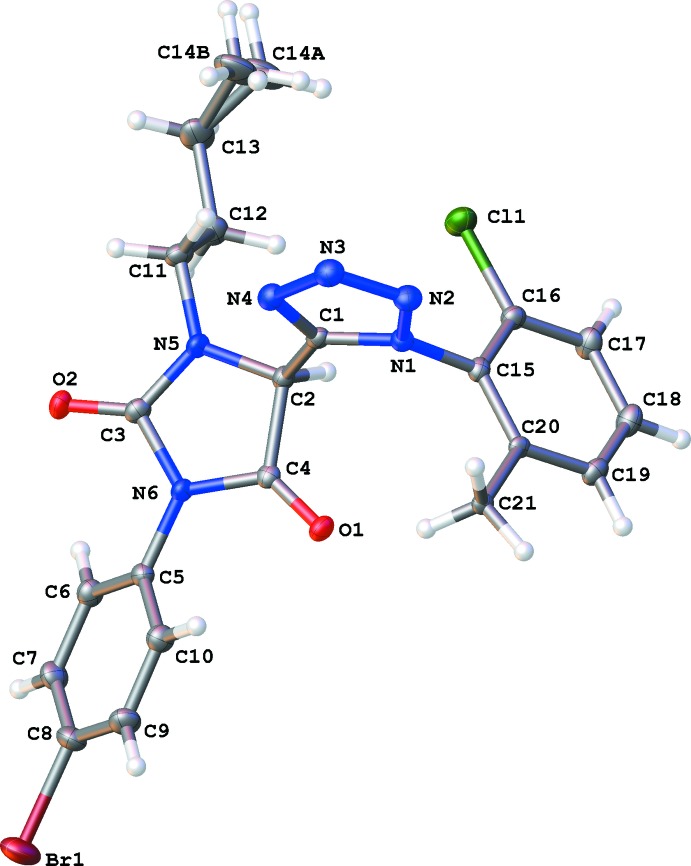
The molecular structure of the title compound. Anisotropically refined atoms are shown as 50% probability ellipsoids.

**Fig. 2.**
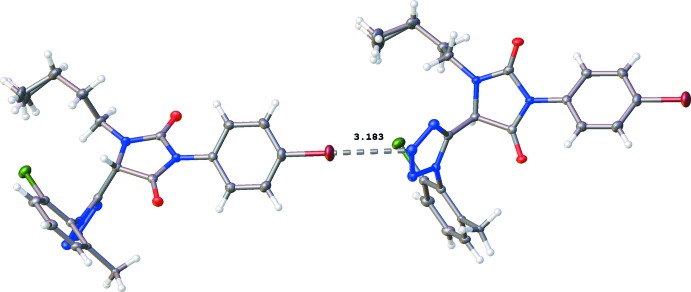
Short contact between Br1 and N3 (3.1834 (1) Å).

## supplementary crystallographic information

### Comment

Hydantoins, also known as imidazoline-2,4-diones, have been shown to display a wide range of biological activities, including anti-convulsant, anti-muscarinic, anti-ulcer, anti-viral and anti-diabetic activities (Thenmozhiyal *et al.*, 2004; Brazil & Pedley, 1998; Luer, 1998; Matzukura *et al.*, 1992; Knabe *et al.*, 1997; Somsák *et al.*, 2001; Moloney *et al.*, 2001; Moloney *et al.*, 1999; Sutherland & Hess, 2000). In an analogous fashion, 1-5-disubstituted tetrazoles are common motifs in a pharmacologically rich vein of chemical space (Davulcu *et al.*, 2009; Al-Hourani *et al.*, 2011; Van Poecke *et al.*, 2011; Quan *et al.*, 2003). In particular, their importance resides in the capacity to act as a bioisosteres of *cis*-amide bonds (Herr, 2002).

The molecular structure is shown in Fig. 1. The terminal carbon of the butyl substituent is disordered between two positions, with occupancies that refine to 0.67 (3) for C14A and 0.33 (3) for C14B. As seen in Fig. 2, a short contact of 3.183 (2) Å is present between Br1 and N3 of a symmetry related tetrazole ring (0.5+x, 1.5-y, 0.5+z) with a C8—Br1—N3 angle of 174.57 (8)°. The van der Waals radii of the interacting atoms sum to 3.40 Å. The plane of the tetrazole ring (C1/N1-N4) makes a dihedral angle of 77.84 (7)° angle with the the plane of the neighboring chloro-substituted benzene ring (C15—C20). The imidazole ring (C2-C4/N5/N6) plane makes a dihedral angle of 43.95 (6)° relative to the plane of the bromo-substituted benzene ring (C5—C10) and the angle between the planes of the tetrazole and imidazole rings is 67.42 (8)°.

### Experimental

Ethyl glyoxalate (50% solution in toluene, 1.10 g, 5.28 mmol, 1 eq) and 1-butylamine (385 mg, 5.28 mmol, 1 eq) were dissolved in dichloroethane (10 ml) in a 35-ml vial and subjected to microwave irradiation at 393 K for 1 h using a CEM initiator. CF_3_CH_2_OH (5 ml) was added, followed by azidotrimethylsilane (TMSN_3_) (610 mg, 5.28 mmol, 1 eq) and 2-chloro-6-methyl-phenylisocianide (797 mg, 5.28 mmol, 1 eq). The resulting mixture was stirred at room temperature for 12 h. After removal of the solvent under reduced pressure, the TMSN_3_—Ugi product was purified by silica gel column chromatography (ethyl acetate-hexane, 0–30%) and isolated as a pale yellow oil (500 mg, 1.42 mmol, 54%). This intermediate (250 mg, 0.80 mmol) was dissolved in dry ethanol (2 ml) under a nitrogen atmosphere. 4-bromo-phenylisocyanate (474 mg, 2.40 mmol, 3 eq) was added, and the reaction stirred at room temperature for 12 h. The title compound precipitated from the reaction mixture and was isolated by filtration as a white microcrystalline solid (220 mg, 0.43 mmol, 77%). Crystals suitable for X-ray crystal structure determination were obtained by slow evaporation of an ethyl acetate-hexane solution of the title compound: Mp 458-461 K.

### Refinement

All hydrogen atoms were visible in a difference Fourier map with the exception of those on the disordered terminal carbon of the butyl group and were added at calculated positions. Hydogen bond distances were set at 0.95 Å for aromatic H atoms, 0.99 Å for alkyl H atoms, and 0.98 Å for methyl H atoms. Themal parameters for all methyl hydrogen atoms were set to 1.50 times the isotropic equivalent thermal parameter of the atom to which they were attached. The thermal parameters of all other hydrogen atoms were set to 1.20 times the isotropic equivalent thermal parameter of the atom to which they were attached.

### Crystal data

**Table:**

C_21_H_20_BrClN_6_O_2_	*F*(000) = 2048
*M**_r_* = 503.79	*D*_x_ = 1.487 Mg m^−^^3^
Monoclinic, *C*2/*c*	Mo *K*α radiation, λ = 0.71073 Å
*a* = 27.9412 (9) Å	Cell parameters from 9862 reflections
*b* = 8.8675 (3) Å	θ = 2.4–27.9°
*c* = 19.6581 (6) Å	µ = 1.98 mm^−^^1^
β = 112.500 (1)°	*T* = 100 K
*V* = 4499.9 (3) Å^3^	Rectangular, colourless
*Z* = 8	0.36 × 0.2 × 0.03 mm

### Data collection

**Table:**

Bruker APEXII DUO CCD diffractometer	5647 independent reflections
Radiation source: fine-focus sealed tube	4475 reflections with *I* > 2σ(*I*)
Graphite monochromator	*R*_int_ = 0.043
φ and ω scans	θ_max_ = 28.5°, θ_min_ = 1.6°
Absorption correction: multi-scan (*SADABS*; Bruker, 2009)	*h* = −37→36
*T*_min_ = 0.613, *T*_max_ = 0.746	*k* = −11→11
79112 measured reflections	*l* = −26→24

### Refinement

**Table:**

Refinement on *F*^2^	Primary atom site location: structure-invariant direct methods
Least-squares matrix: full	Secondary atom site location: difference Fourier map
*R*[*F*^2^ > 2σ(*F*^2^)] = 0.037	Hydrogen site location: inferred from neighbouring sites
*wR*(*F*^2^) = 0.100	H-atom parameters not refined
*S* = 1.03	*w* = 1/[σ^2^(*F*_o_^2^) + (0.0424*P*)^2^ + 14.0493*P*] where *P* = (*F*_o_^2^ + 2*F*_c_^2^)/3
5647 reflections	(Δ/σ)_max_ = 0.013
281 parameters	Δρ_max_ = 1.34 e Å^−^^3^
12 restraints	Δρ_min_ = −1.07 e Å^−^^3^

### Special details

**Table:**

Geometry. All e.s.d.'s (except the e.s.d. in the dihedral angle between two l.s. planes) are estimated using the full covariance matrix. The cell e.s.d.'s are taken into account individually in the estimation of e.s.d.'s in distances, angles and torsion angles; correlations between e.s.d.'s in cell parameters are only used when they are defined by crystal symmetry. An approximate (isotropic) treatment of cell e.s.d.'s is used for estimating e.s.d.'s involving l.s. planes.
Refinement. Refinement of *F*^2^ against ALL reflections. The weighted *R*-factor *wR* and goodness of fit *S* are based on *F*^2^, conventional *R*-factors *R* are based on *F*, with *F* set to zero for negative *F*^2^. The threshold expression of *F*^2^ > σ(*F*^2^) is used only for calculating *R*-factors(gt) *etc*. and is not relevant to the choice of reflections for refinement. *R*-factors based on *F*^2^ are statistically about twice as large as those based on *F*, and *R*-factors based on ALL data will be even larger.

### Fractional atomic coordinates and isotropic or equivalent isotropic displacement parameters (Å^2^)

**Table:**

	*x*	*y*	*z*	*U*_iso_*/*U*_eq_	Occ. (<1)
Br1	1.011728 (9)	0.62404 (4)	0.390312 (15)	0.03786 (10)	
Cl1	0.57351 (2)	0.43637 (7)	0.01505 (3)	0.02585 (13)	
O1	0.77362 (6)	0.54096 (17)	0.10987 (8)	0.0178 (3)	
N6	0.77924 (7)	0.65177 (19)	0.21947 (9)	0.0146 (3)	
N1	0.65391 (7)	0.62551 (19)	−0.00380 (9)	0.0140 (3)	
O2	0.75496 (6)	0.77690 (18)	0.30583 (8)	0.0201 (3)	
N5	0.69524 (7)	0.6804 (2)	0.19766 (10)	0.0167 (4)	
N4	0.65662 (7)	0.8290 (2)	0.05861 (10)	0.0178 (4)	
C8	0.93903 (9)	0.6319 (3)	0.33660 (13)	0.0239 (5)	
C7	0.90634 (9)	0.5992 (3)	0.37241 (12)	0.0222 (5)	
H7	0.9201	0.5716	0.4229	0.027*	
C6	0.85319 (9)	0.6071 (2)	0.33357 (12)	0.0188 (4)	
H6	0.8301	0.5844	0.3572	0.023*	
C5	0.83404 (8)	0.6485 (2)	0.25987 (11)	0.0151 (4)	
C4	0.75450 (8)	0.5905 (2)	0.15058 (11)	0.0142 (4)	
C2	0.69640 (8)	0.6013 (2)	0.13411 (11)	0.0146 (4)	
H2	0.6813	0.4981	0.1311	0.018*	
C1	0.66895 (8)	0.6853 (2)	0.06412 (11)	0.0137 (4)	
C15	0.65419 (8)	0.4734 (2)	−0.02730 (11)	0.0143 (4)	
C16	0.61720 (8)	0.3741 (2)	−0.02185 (11)	0.0160 (4)	
C17	0.61567 (9)	0.2265 (2)	−0.04531 (12)	0.0207 (4)	
H17	0.5907	0.1579	−0.0414	0.025*	
C18	0.65116 (9)	0.1805 (3)	−0.07466 (12)	0.0213 (4)	
H18	0.6503	0.0795	−0.0912	0.026*	
C10	0.86722 (9)	0.6800 (3)	0.22433 (12)	0.0201 (4)	
H10	0.8537	0.7077	0.1738	0.024*	
C9	0.92031 (9)	0.6708 (3)	0.26306 (13)	0.0239 (5)	
H9	0.9435	0.6911	0.2393	0.029*	
C3	0.74322 (8)	0.7112 (2)	0.24774 (12)	0.0160 (4)	
N2	0.63105 (7)	0.7362 (2)	−0.05322 (10)	0.0176 (4)	
N3	0.63295 (7)	0.8568 (2)	−0.01536 (11)	0.0197 (4)	
C19	0.68770 (8)	0.2792 (2)	−0.08031 (11)	0.0178 (4)	
H19	0.7118	0.2447	−0.1003	0.021*	
C20	0.68992 (7)	0.4282 (2)	−0.05726 (10)	0.0122 (3)	
C11	0.64797 (8)	0.7115 (3)	0.21019 (12)	0.0190 (4)	
H11A	0.6218	0.7550	0.1649	0.023*	
H11B	0.6555	0.7871	0.2500	0.023*	
C12	0.62577 (9)	0.5705 (3)	0.23131 (13)	0.0228 (5)	
H12A	0.6514	0.5293	0.2778	0.027*	
H12B	0.6196	0.4931	0.1925	0.027*	
C13	0.57507 (10)	0.6024 (3)	0.24118 (14)	0.0316 (6)	
H13A	0.5685	0.5205	0.2707	0.038*	0.67 (3)
H13B	0.5784	0.6979	0.2687	0.038*	0.67 (3)
H13C	0.5822	0.6736	0.2827	0.038*	0.33 (3)
H13D	0.5625	0.5072	0.2548	0.038*	0.33 (3)
C14A	0.5291 (4)	0.614 (2)	0.1675 (7)	0.046 (2)	0.67 (3)
H14A	0.5353	0.6959	0.1383	0.069*	0.67 (3)
H14B	0.4975	0.6356	0.1763	0.069*	0.67 (3)
H14C	0.5250	0.5188	0.1407	0.069*	0.67 (3)
C14B	0.5321 (10)	0.668 (3)	0.1735 (16)	0.046 (2)	0.33 (3)
H14D	0.5426	0.7676	0.1626	0.069*	0.33 (3)
H14E	0.5003	0.6774	0.1831	0.069*	0.33 (3)
H14F	0.5258	0.6009	0.1313	0.069*	0.33 (3)
C21	0.73027 (7)	0.5400 (2)	−0.06540 (10)	0.0122 (3)	
H21A	0.7124	0.6299	−0.0922	0.018*	
H21B	0.7489	0.4912	−0.0927	0.018*	
H21C	0.7549	0.5695	−0.0165	0.018*	

### Atomic displacement parameters (Å^2^)

**Table:**

	*U*^11^	*U*^22^	*U*^33^	*U*^12^	*U*^13^	*U*^23^
Br1	0.01517 (12)	0.0592 (2)	0.03350 (15)	0.00097 (11)	0.00299 (10)	−0.00200 (13)
Cl1	0.0240 (3)	0.0277 (3)	0.0311 (3)	−0.0026 (2)	0.0164 (2)	−0.0040 (2)
O1	0.0192 (7)	0.0203 (7)	0.0158 (7)	0.0027 (6)	0.0089 (6)	−0.0023 (6)
N6	0.0153 (8)	0.0169 (8)	0.0131 (8)	0.0000 (7)	0.0071 (7)	−0.0021 (7)
N1	0.0143 (8)	0.0136 (8)	0.0140 (8)	0.0004 (6)	0.0052 (7)	0.0007 (7)
O2	0.0221 (8)	0.0236 (8)	0.0166 (7)	−0.0013 (6)	0.0096 (6)	−0.0064 (6)
N5	0.0166 (8)	0.0209 (9)	0.0144 (8)	0.0002 (7)	0.0080 (7)	−0.0034 (7)
N4	0.0196 (9)	0.0148 (8)	0.0204 (9)	−0.0006 (7)	0.0093 (7)	0.0001 (7)
C8	0.0136 (10)	0.0303 (12)	0.0247 (11)	−0.0003 (9)	0.0038 (9)	−0.0032 (10)
C7	0.0200 (10)	0.0287 (12)	0.0149 (10)	0.0007 (9)	0.0032 (8)	−0.0003 (9)
C6	0.0205 (10)	0.0210 (10)	0.0164 (10)	−0.0014 (8)	0.0089 (8)	−0.0004 (8)
C5	0.0142 (9)	0.0156 (9)	0.0154 (10)	−0.0002 (7)	0.0054 (8)	−0.0016 (8)
C4	0.0162 (9)	0.0119 (9)	0.0142 (9)	0.0017 (7)	0.0057 (8)	0.0016 (7)
C2	0.0156 (9)	0.0156 (9)	0.0139 (9)	0.0000 (7)	0.0071 (8)	−0.0012 (8)
C1	0.0128 (9)	0.0137 (9)	0.0166 (10)	−0.0007 (7)	0.0076 (8)	−0.0005 (8)
C15	0.0166 (9)	0.0131 (9)	0.0124 (9)	0.0015 (7)	0.0047 (8)	0.0003 (7)
C16	0.0158 (9)	0.0188 (10)	0.0150 (9)	0.0003 (8)	0.0076 (8)	0.0000 (8)
C17	0.0265 (11)	0.0166 (10)	0.0224 (11)	−0.0049 (9)	0.0133 (9)	−0.0014 (9)
C18	0.0305 (12)	0.0156 (10)	0.0202 (11)	0.0005 (9)	0.0123 (9)	−0.0004 (8)
C10	0.0202 (10)	0.0230 (11)	0.0178 (10)	0.0000 (9)	0.0082 (9)	0.0004 (9)
C9	0.0185 (11)	0.0313 (12)	0.0251 (12)	−0.0021 (9)	0.0121 (9)	−0.0012 (10)
C3	0.0178 (10)	0.0167 (10)	0.0165 (10)	0.0004 (8)	0.0098 (8)	0.0016 (8)
N2	0.0184 (8)	0.0164 (8)	0.0177 (9)	0.0026 (7)	0.0065 (7)	0.0042 (7)
N3	0.0209 (9)	0.0168 (9)	0.0225 (9)	0.0010 (7)	0.0096 (8)	0.0029 (7)
C19	0.0223 (10)	0.0190 (10)	0.0151 (10)	0.0031 (8)	0.0105 (8)	0.0007 (8)
C20	0.0127 (6)	0.0166 (7)	0.0085 (6)	0.0002 (5)	0.0054 (5)	0.0007 (5)
C11	0.0166 (10)	0.0238 (11)	0.0197 (10)	0.0024 (8)	0.0104 (8)	0.0002 (9)
C12	0.0230 (11)	0.0280 (12)	0.0209 (11)	−0.0025 (9)	0.0123 (9)	−0.0014 (9)
C13	0.0252 (12)	0.0470 (16)	0.0291 (13)	−0.0084 (11)	0.0176 (11)	−0.0046 (12)
C14A	0.0167 (19)	0.079 (8)	0.043 (3)	−0.011 (4)	0.0123 (19)	−0.004 (5)
C14B	0.0167 (19)	0.079 (8)	0.043 (3)	−0.011 (4)	0.0123 (19)	−0.004 (5)
C21	0.0127 (6)	0.0166 (7)	0.0085 (6)	0.0002 (5)	0.0054 (5)	0.0007 (5)

### Geometric parameters (Å, º)

**Table:**

Br1—C8	1.899 (2)	C18—H18	0.9500
Cl1—C16	1.732 (2)	C18—C19	1.381 (3)
O1—C4	1.203 (2)	C10—H10	0.9500
N6—C5	1.430 (3)	C10—C9	1.387 (3)
N6—C4	1.375 (3)	C9—H9	0.9500
N6—C3	1.424 (3)	N2—N3	1.292 (3)
N1—C1	1.346 (3)	C19—H19	0.9500
N1—C15	1.427 (3)	C19—C20	1.390 (3)
N1—N2	1.356 (2)	C20—C21	1.555 (3)
O2—C3	1.210 (3)	C11—H11A	0.9900
N5—C2	1.444 (3)	C11—H11B	0.9900
N5—C3	1.353 (3)	C11—C12	1.522 (3)
N5—C11	1.459 (3)	C12—H12A	0.9900
N4—C1	1.314 (3)	C12—H12B	0.9900
N4—N3	1.370 (3)	C12—C13	1.528 (3)
C8—C7	1.380 (3)	C13—H13A	0.9900
C8—C9	1.380 (3)	C13—H13B	0.9900
C7—H7	0.9500	C13—H13C	0.9900
C7—C6	1.388 (3)	C13—H13D	0.9900
C6—H6	0.9500	C13—C14A	1.527 (12)
C6—C5	1.388 (3)	C13—C14B	1.53 (3)
C5—C10	1.386 (3)	C14A—H14A	0.9800
C4—C2	1.532 (3)	C14A—H14B	0.9800
C2—H2	1.0000	C14A—H14C	0.9800
C2—C1	1.493 (3)	C14B—H14D	0.9800
C15—C16	1.393 (3)	C14B—H14E	0.9800
C15—C20	1.398 (3)	C14B—H14F	0.9800
C16—C17	1.383 (3)	C21—H21A	0.9800
C17—H17	0.9500	C21—H21B	0.9800
C17—C18	1.386 (3)	C21—H21C	0.9800
			
C4—N6—C5	124.55 (17)	C10—C9—H9	120.4
C4—N6—C3	111.50 (17)	O2—C3—N6	124.71 (19)
C3—N6—C5	123.86 (17)	O2—C3—N5	128.24 (19)
C1—N1—C15	130.94 (17)	N5—C3—N6	107.04 (17)
C1—N1—N2	107.96 (16)	N3—N2—N1	106.38 (16)
N2—N1—C15	120.77 (16)	N2—N3—N4	111.07 (17)
C2—N5—C11	123.99 (17)	C18—C19—H19	119.4
C3—N5—C2	112.53 (17)	C18—C19—C20	121.21 (19)
C3—N5—C11	123.23 (17)	C20—C19—H19	119.4
C1—N4—N3	105.46 (17)	C15—C20—C21	121.55 (18)
C7—C8—Br1	118.98 (18)	C19—C20—C15	117.37 (18)
C9—C8—Br1	119.19 (17)	C19—C20—C21	121.08 (17)
C9—C8—C7	121.8 (2)	N5—C11—H11A	109.1
C8—C7—H7	120.4	N5—C11—H11B	109.1
C8—C7—C6	119.1 (2)	N5—C11—C12	112.39 (18)
C6—C7—H7	120.4	H11A—C11—H11B	107.9
C7—C6—H6	120.3	C12—C11—H11A	109.1
C5—C6—C7	119.4 (2)	C12—C11—H11B	109.1
C5—C6—H6	120.3	C11—C12—H12A	109.2
C6—C5—N6	119.21 (18)	C11—C12—H12B	109.2
C6—C5—C10	121.0 (2)	C11—C12—C13	112.1 (2)
C10—C5—N6	119.76 (19)	H12A—C12—H12B	107.9
O1—C4—N6	128.02 (19)	C13—C12—H12A	109.2
O1—C4—C2	125.92 (19)	C13—C12—H12B	109.2
N6—C4—C2	106.04 (16)	C12—C13—H13A	109.2
N5—C2—C4	102.73 (16)	C12—C13—H13B	109.2
N5—C2—H2	110.2	C12—C13—H13C	108.6
N5—C2—C1	112.39 (17)	C12—C13—H13D	108.6
C4—C2—H2	110.2	C12—C13—C14B	114.7 (10)
C1—C2—C4	110.81 (16)	H13A—C13—H13B	107.9
C1—C2—H2	110.2	H13C—C13—H13D	107.6
N1—C1—C2	124.98 (18)	C14A—C13—C12	112.1 (5)
N4—C1—N1	109.13 (18)	C14A—C13—H13A	109.2
N4—C1—C2	125.88 (19)	C14A—C13—H13B	109.2
C16—C15—N1	118.47 (18)	C14B—C13—H13C	108.6
C16—C15—C20	121.44 (19)	C14B—C13—H13D	108.6
C20—C15—N1	120.05 (18)	C13—C14A—H14A	109.5
C15—C16—Cl1	119.55 (16)	C13—C14A—H14B	109.5
C17—C16—Cl1	120.31 (16)	C13—C14A—H14C	109.5
C17—C16—C15	120.14 (19)	C13—C14B—H14D	109.5
C16—C17—H17	120.6	C13—C14B—H14E	109.5
C18—C17—C16	118.8 (2)	C13—C14B—H14F	109.5
C18—C17—H17	120.6	H14D—C14B—H14E	109.5
C17—C18—H18	119.5	H14D—C14B—H14F	109.5
C19—C18—C17	121.1 (2)	H14E—C14B—H14F	109.5
C19—C18—H18	119.5	C20—C21—H21A	109.5
C5—C10—H10	120.2	C20—C21—H21B	109.5
C5—C10—C9	119.5 (2)	C20—C21—H21C	109.5
C9—C10—H10	120.2	H21A—C21—H21B	109.5
C8—C9—C10	119.1 (2)	H21A—C21—H21C	109.5
C8—C9—H9	120.4	H21B—C21—H21C	109.5
			
Br1—C8—C7—C6	179.01 (17)	C1—N1—C15—C20	−107.9 (2)
Br1—C8—C9—C10	−178.49 (18)	C1—N1—N2—N3	0.3 (2)
Cl1—C16—C17—C18	179.91 (17)	C1—N4—N3—N2	−0.2 (2)
O1—C4—C2—N5	−175.0 (2)	C15—N1—C1—N4	−173.71 (19)
O1—C4—C2—C1	−54.7 (3)	C15—N1—C1—C2	7.6 (3)
N6—C5—C10—C9	−177.4 (2)	C15—N1—N2—N3	174.40 (18)
N6—C4—C2—N5	3.6 (2)	C15—C16—C17—C18	−0.5 (3)
N6—C4—C2—C1	123.80 (18)	C16—C15—C20—C19	−1.1 (3)
N1—C15—C16—Cl1	−1.6 (3)	C16—C15—C20—C21	178.28 (18)
N1—C15—C16—C17	178.84 (19)	C16—C17—C18—C19	0.3 (3)
N1—C15—C20—C19	−178.92 (18)	C17—C18—C19—C20	−0.4 (3)
N1—C15—C20—C21	0.4 (3)	C18—C19—C20—C15	0.8 (3)
N1—N2—N3—N4	−0.1 (2)	C18—C19—C20—C21	−178.54 (19)
N5—C2—C1—N1	−164.34 (18)	C9—C8—C7—C6	−0.7 (4)
N5—C2—C1—N4	17.2 (3)	C3—N6—C5—C6	42.6 (3)
N5—C11—C12—C13	−177.58 (19)	C3—N6—C5—C10	−140.3 (2)
C8—C7—C6—C5	−0.4 (3)	C3—N6—C4—O1	174.4 (2)
C7—C8—C9—C10	1.2 (4)	C3—N6—C4—C2	−4.1 (2)
C7—C6—C5—N6	177.97 (19)	C3—N5—C2—C4	−1.9 (2)
C7—C6—C5—C10	0.9 (3)	C3—N5—C2—C1	−121.07 (19)
C6—C5—C10—C9	−0.4 (3)	C3—N5—C11—C12	−101.3 (2)
C5—N6—C4—O1	−9.1 (3)	N2—N1—C1—N4	−0.5 (2)
C5—N6—C4—C2	172.39 (18)	N2—N1—C1—C2	−179.10 (18)
C5—N6—C3—O2	6.8 (3)	N2—N1—C15—C16	−98.3 (2)
C5—N6—C3—N5	−173.55 (18)	N2—N1—C15—C20	79.6 (2)
C5—C10—C9—C8	−0.7 (4)	N3—N4—C1—N1	0.4 (2)
C4—N6—C5—C6	−133.4 (2)	N3—N4—C1—C2	179.02 (18)
C4—N6—C5—C10	43.7 (3)	C20—C15—C16—Cl1	−179.49 (16)
C4—N6—C3—O2	−176.7 (2)	C20—C15—C16—C17	1.0 (3)
C4—N6—C3—N5	2.9 (2)	C11—N5—C2—C4	−176.40 (18)
C4—C2—C1—N1	81.4 (2)	C11—N5—C2—C1	64.5 (3)
C4—C2—C1—N4	−97.0 (2)	C11—N5—C3—N6	174.12 (18)
C2—N5—C3—N6	−0.4 (2)	C11—N5—C3—O2	−6.3 (4)
C2—N5—C3—O2	179.2 (2)	C11—C12—C13—C14A	78.3 (8)
C2—N5—C11—C12	72.6 (3)	C11—C12—C13—C14B	58.3 (12)
C1—N1—C15—C16	74.2 (3)		
